# Work-related experiences of prostate cancer survivors in Australia: a qualitative study

**DOI:** 10.1186/s12889-023-16706-4

**Published:** 2023-09-16

**Authors:** Wei-Hong Liu, Jennifer Fox, Patsy Yates

**Affiliations:** 1 Cancer and Palliative Care Outcomes Centre, Faculty of Health, Queensland University of Technology, Brisbane, QLD Australia; 2 Cancer and Palliative Care Outcomes Centre, Faculty of Health, Queensland University of Technology, Kelvin Grove, QLD 4059 Australia

**Keywords:** Cancer (Neoplasms), Employment, Prostate, Qualitative, Self-employed, Survivorship, Work

## Abstract

**Background:**

Prostate cancer (PCa) is the most diagnosed cancer in Australian men, and the number of survivors is growing with advances in diagnosis and treatment. Work participation following PCa diagnosis and treatment becomes a significant aspect of quality of life and survivorship. Using a qualitative phenomenological approach, we explored the work-related experiences of PCa survivors in Australia.

**Methods:**

Semi-structured telephone interviews were conducted with 16 men (6 salaried employees, 10 self-employed; 8 diagnosed ≥ 5 years) purposively sampled from a community setting. Interviews were inductively analysed.

**Results:**

Five main themes emerged: motivations to work; treatment decisions and work; the effects of PCa and its treatment on ability to participate in work; being an employee versus being self-employed; and personal agency. PCa and its treatment side-effects were detrimental to men’s work capacity and ability, and could persist over an extended period. Most men expressed a strong desire to retain work or return to work. Discussions with healthcare professionals about work-related consequences were largely missing when treatment decisions were made. Self-employed men faced greater challenges than their salaried counterparts due to high financial burden and limited social and business support. Family, workplace and wider community support, and self-care, enhanced men’s work participation experiences.

**Conclusions:**

PCa and its treatment substantially and persistently impacted men’s working lives, and their experiences were diverse and multifaceted. Self-employed and long-term PCa survivors face greater challenges and are at high risk of poor work outcomes. A systematic approach and involvement of stakeholders at all levels is required to support ongoing work participation.

## Background

The annual cost of lost labour force participation by people who have been diagnosed with any form of cancer accounts for approximately $1.7 billion in gross domestic product to the Australian economy [1]. The most common cancer in Australian men, prostate cancer (PCa), affects around 20,000 men each year, of which nearly 40% are under the age of 65 [2]. The likelihood of men receiving a PCa diagnosis while still working is increasing due to the growing number of men who remain in the labour force beyond the age of 65 years [3]. Given the high survival rate of men with PCa in Australia (95.5%) [2], work participation becomes an important aspect of PCa survivorship.

There is a consensus that work participation after a cancer diagnosis is beneficial for both individuals and society, such as enhancing psychosocial well-being, promoting financial independence, and reducing loss of productivity [4–7]. Work for PCa survivors has been shown to be vital for their masculine identities, sense of personal growth and control, and financial security [8, 9]. However, continuing to work or returning to work after a PCa diagnosis can be challenging. Overall, 60–90% men in economically developed countries return to work and the majority return to work within the first year after the PCa diagnosis [10]. Yet the risk of early retirement due to ill health is higher in men with PCa compared with men with other types of cancers [10]. Compared with those without cancer, men with PCa were 39% more likely to retire due to ill health and men with non-localised PCa were 21% more likely to be out of the workforce [11]. Adjusting for age, PCa survivors who were diagnosed within the last 5 years were 4% more likely to be out of the workforce, but 14% more likely to be out of the workforce if they had been diagnosed for 5 or more years [11].

Work participation after a cancer diagnosis can be a complex process affected by many factors [7, 12, 13]. Factors relating to the individual that are health-related, such as symptoms and functions, and non-health related, such as age, socioeconomic status, family, social supports, and coping strategies, all affect outcomes. Work-related factors, such as work demands and work environment, as well as general organisational, legal, and financial factors also contribute. It is well documented that PCa and its treatment result in a range of short-term and long-term adverse effects on men’s physical, cognitive, and psychosocial functions which significantly impede men’s work capacity and ability [7, 8, 10]. Previous studies have primarily used quantitative approaches to examine these effects on short-term (< 5 years post-diagnosis) work-related outcomes, including return-to-work rate, sick leave and time taken to return-to-work, reductions in work hours and income, and incidence of early retirement [10]. However, the extent to which these consequences negatively impact men’s work decisions and subsequent return-to-work processes is less understood. The problems men experience related to working after a PCa diagnosis may differ across their survivorship journey. Moreover, it remains unclear how non-health related and/or other broader contextual factors affect the working experience of men with PCa. Given these gaps in the research, qualitative approaches to explore work-related needs and experiences of men with PCa can contribute to our understanding of men’s needs in this area [7, 10].

This study explores the work-related experience of PCa survivors in Australia. Our specific research questions were: 1) How did the PCa diagnosis and related treatments impact their work participation? 2) What were the work-related needs of men when they maintained their work or returned to work at any stage of their cancer journey? 3) How did men cope with work-related challenges after a PCa diagnosis?

## Methods

This study adopted a qualitative phenomenological approach to explore work-related experiences of men affected by PCa. This approach allows the researcher to gain insight into the perspectives of participants without prejudice [14]. The Consolidated Criteria for Reporting Qualitative Research (COREQ) guidelines [15] were followed in reporting the study findings.

### Participants

Participants of the study were men aged over 18 years, able to read and speak English, and were either in paid employment or self-employed at the time of being diagnosed with PCa. Using a purposive sampling strategy, PCa community support groups across Australia were identified on the internet and by word of mouth. The researcher (WHL) approached the contact person or group leaders of these groups via email between March and June 2020. They were asked to distribute a recruitment flyer to members of the groups via newsletters or email. Written consent was obtained from eligible men who contacted the researcher (WHL) and expressed their interest in the study. The study was approved by the Human Research Ethics Committee of Queensland University of Technology (Approval number: 1900000433).

### Data collection

The first author and two research assistants who were trained and experienced in interviews with cancer patients conducted one-to-one semi-structured telephone interviews with the participants. General information about the participants such as age, socioeconomic status, work and PCa related information, was collected over the phone at the beginning of each interview. The interview was then conducted using an interview guide and was digitally recorded. Table 1 shows example interview questions.

**Table 1:** Examples of interview questions

• What was work like before PCa? How did PCa diagnosis or treatment affect your work?
• What were the barriers preventing you from going back to work on a regular and sustainable basis?
• What strategies did you use to overcome any challenges you encountered?
• What support would enable you to work whilst undergoing treatments or to return to work after treatments?

### Data analysis

The audio recordings of the interviews were transcribed verbatim by a professional transcriber and one of the research assistants. The length of the interviews varied from 15 to 45 min. An inductive approach [16] was used to analyse the transcripts, in which themes and explanations were derived primarily from a close reading of the data, without trying to fit the data to pre-existing concepts or ideas from theory. This process included being immersed in the data by listening to the recorded interviews and by reading and re-reading the transcripts, as well as by coding the texts, categorising similar meaning units, spelling out each meaning unit, and by combining these into thematic statements. Two authors (WL and JF) each coded the interviews separately and differences in researcher perspectives were discussed until mutual agreement was reached.

## Results

The recruitment occurred mostly in March and April 2020, at the beginning of the national lockdown due to the Covid-19 pandemic. A total of 29 eligible men contacted the researcher and expressed interest in the study. However, a few men later explained that there was too much going on in their lives, and some men did not respond to further contact. As a result, 16 men consented and participated in the interview. The characteristics of the participants are summarised in Table 2. The median age of the men was 68 years (range 55 to 81) and eight of the 16 men had been diagnosed with PCa for five years or longer. At the time of diagnosis, all men were in the workforce; 10 were self-employed and 11 were under 65. At the time of the study, 10 men were retired due to ill health (*n* = 3) or aging/non-health related reasons (*n* = 7), three (≤ 60) were working full- or part-time, one (> 65) was employed on a casual basis, and two (one of them > 65) were unemployed and actively seeking work. Of the 10 self-employed men, only two were still in the workforce at interview. One of them changed to paid employment, and another one changed from working full-time to part-time.

**Table 2:** The characteristics of study participants (*n* = 16)

**Participant**	**Age group** (years)	**Length of diagnosis** (years)	**Stage of PCa** Diagnosis → Current	**Treatment received**	**Employment status** Diagnosis → Current	**Occupation**	**No. of employees at workplace**
1	65–69	< 1	Adv → Adv	S	Salaried → Salaried	Professional	> 200
2	70–74	10–14	Loc → Adv	S, H, C	Self → Retired	Manager	< 10
3	65–69	15–19	Loc → Adv	S, R, H	Salaried → Unemployed	Professional	> 200
4	60–64	1–2	Loc → Loc	R	Self → Salaried	Professional	0
5	60–64	6–9	Adv → Adv	S, R, H	Self → Retired	Trade worker	< 10
6	75–79	3–5	Adv → Adv	C, R	Self → Retired	Manager	50–199
7	55–59	3–5	Adv → Adv	R, H, C	Self → Self	Professional	0
8	55–59	1–2	Loc → Loc	A	Self → Unemployed	Trade worker	< 10
9	70–74	3–5	Loc → Loc	S	Salaried → Retired	Professional	20–49
10	80–85	15–19	Loc → Loc	R, H	Self → Retired	Professional	< 10
11	55–59	3–5	Loc → Loc	S	Self → Self	Professional	< 10
12	60–64	6–9	Loc → Loc	S	Salaried → Retired	Professional	50–199
13	70–74	3–5	Adv → Adv	C, H	Self → Retired	Manager	20–49
14	70–74	15–19	Loc → Loc	S	Self → Retired	Technician	< 10
15	60–64	1–2	Loc → Loc	S, R	Salaried → Retired	Professional	> 200
16	70–74	6–9	Loc → Loc	S, R	Salaried → Retired	Professional	50–199

*Adv* Advanced, *Loc* Localised, *S* Surgery, *H* Hormone therapy, *R* Radiation therapy, *C* Chemotherapy, *A* Active surveillance, *Self* Self-employed

Five main themes emerged from the data: (1) motivations to work; (2) treatment decisions and work; (3) the effects of prostate cancer and its treatment on ability to participate in work; (4) being an employee versus being self-employed; and (5) personal agency.

### Motivations to work

Men recalled their perspectives on work before their PCa diagnosis. Some described work as being “quite satisfying” (P1), and “the ambition and the zest for life” (P2); others expressed that work provided financial independence and made them “feel better about myself” (P7); a few men noted their work was “high pressure” (P9) and/or endured for “long hours” (P15). For most, the PCa diagnosis did not change their attitude towards work, and they were determined to resume their usual work as soon as possible. In fact, all men noted that they continued working after the diagnosis for various lengths of time.

*There’s nothing to stop me. Health wise there’s nothing to stop me.* (P1)

*I’ve always got to have a project, always got to have something ahead of me.* (P2)

Nonetheless, the majority of men prioritised family and/or health over work following the diagnosis. They explained that “family comes number one to me” (P4) and “a more balanced lifestyle” (P9) was a priority. They discussed the benefits of work as a distraction from cancer and providing “normality and routine” (P7) throughout their treatment. For a few, work was “a necessary evil” (P4) for them to pay off a mortgage and out-of-pocket healthcare expenditure and provide support to their families. On the other hand, one man disclosed that he “didn’t like work” (P16) although he continued working until reaching retirement age.

### Treatment decisions and work

Achieving an optimal health outcome was the primary concern for most men when making treatment decisions for their PCa, demonstrated by comments such as “it was the decision made purely on what I considered was the best option for treatment” (P2) and “I don’t think it [work] was at the front of my mind at all” (P9). Only a few men considered how treatment would potentially impact their work, and even then, they perceived their health to be more important than continuing to work. Several men expressed their preference for less invasive treatment or treatment that had minimal impact on their work and/or lifestyles.

*That was a consideration how could I afford to take half a day off to get some treatment and then what would be the side-effects after that. Which would mean further time off doing it, but the Urologist recommended the radical because the condition of the cancer.* (P12)

*I had a form of treatment that enabled me to go back to work for another 10 years.* (P14)

The men collectively viewed that health professionals are responsible for provision of information about treatment options although some men also sought their own information from a variety of sources, such as the internet, their families, support groups or friends, or health professionals who were not involved in their care. The majority of men reported that they received adequate information regarding treatment options, but some felt that the risks of developing short- and long-term side-effects were not discussed sufficiently, and there was no discussion about how certain types of treatments may affect their ability to work. Men who felt they were not given adequate information about side-effects reported that they struggled with unexpected adverse effects when they did occur, resulting in a negative impact on their ability to work.

*The surgeon I had was very good and straight forward with the facts of what was going to happen, and he was very supportive and freely gave me information and really gave me the confidence … No [discussion about work], not really, it was only advised to take it a bit easier with the lifting until things had healed properly.* (P2)

*I don’t feel like I was adequately informed around the impacts of treatment, particularly the hormone therapy … I didn’t feel adequately prepared for the way the hormone therapy affected me. And it was sort of quite cumulative, so it sort of crept up on me gradually and I mean at its worst I found it just completely debilitating to the point where I was quite depressed … It affects your cognitive function so I felt like I couldn’t [work].* (P7)

Most participants expressed they had confidence in the health professionals to make the right decisions for them. A few participants noted that men should consider their own perspectives and engage actively in decision-making about treatment and its impact on their life, such as what work adjustments are required during and after treatment, and how side-effects of treatment impact their quality of life and capacity to work. On the other hand, not all men at diagnosis had options for alternative treatment. The decision was left up to them to choose, quickly, between what the doctors felt would give them the best chance of survival.

*I just don’t think any man gets out scot-free, they really need to consider, in my opinion they need to consider what options they have … People have got different priorities.* (P9)

*They said, ‘One more week and it’s probably in your bones’. So I was very lucky that it wasn’t in my bones and they said, ‘If you don’t do anything you’ll be dead in six months’. That happened over the first three months because they loaded me up with all these medications that sent me spiralling out of control. … I couldn’t go to work. … I was wrecked.* (P5)

### The effects of prostate cancer and its treatment on ability to participate in work

The diagnosis of PCa and/or its treatments interrupted the men’s work in different ways depending on types of treatments received, and on occupation. For example, the type of industry, size of business, and position at work were all factors. Some men continued to work until just before treatment, while others were able to continue work during treatment or return to work after treatment. Nearly all men reported they experienced some cancer-related and/or treatment-related symptoms of varying severity and functional impairment, which adversely affected their daily lives and functioning, including work. For most men, incontinence and fatigue following treatments were the main health problems affecting their ability to perform their usual roles.

*The only thing that might have prevented me from going back to work is the incontinence.* (P1)

*The fatigue [is] like that, it’s not just physical, because it affects your mental processes as well. After a while you find you can’t think clearly, so you’re not in the right space for making proper management decisions and that sort of thing. So that has a direct impact on your ability to work.* (P15)

Some men perceived that they had been “lucky”, or “fortunate” and their own experiences were less severe than others. They described that their treatment and recovery occurred within a certain period from several weeks to several months and proceeded at a pace that enabled them to recalibrate and establish a “new normal” and continue to work.

*A lot of the people that I went through the chemo sessions with had very significant side effects and I was fortunate that I had very little in terms of side effects. … So it didn’t really impact me at all in that respect. … I mean normally you would retire around about fifty-five to seventy and I was still working when I was seventy-nine.* (P6)

#### A lengthy process of adjustment

The men’s ability to work was greatly affected by fluctuations in their health status due to persistent side-effects, and/or the cancer progression experienced over time, and/or secondary treatments. As a result, their work participation sometimes became intermittent, and the recovery and adjustment were a lengthy process.

*I did the [first] work for about eighteen months. I took a break for about six months and then I got the opportunity to [do the second work] ... I’d had a break from the hormone therapy at the time I started [the second work], so I was sort of feeling a lot better and financially I’d sort of, I needed to be working … When I finished the project and I’d had to go back on the medication that I felt like I really sort of hit the wall and went into a bit of a slump.* (P7)

One man who was initially diagnosed with localised PCa shared stories of dealing with cancer progression, side-effects, ageing and comorbidities through extended treatments while continuing to work over more than a decade.

[After initial surgical treatment]* Post-surgery information like mental support and that sort of thing was almost non-existent at that time and that is an area that I struggled with and because radical prostatectomy really upsets you in general … I came out still had all the post op dressings and catheter and the whole works and I was back on the job.* (P2)

*A little bit of incontinence … I think it’s been on hormone treatment for so long its upsets the muscle tones.* (P2)

*[Recent chemotherapy] has [impacted on my ability to work] to a degree … sometimes post the treatment I’m fine for three or four days and then I have a bit of a spell that’s not so good, other times I have that spell right on top of the treatment and then I come good again … Because I’m 74, I’ve got cataracts and the chemo is upsetting the cataracts so that’s causing them a bit of grief at the moment … that has impacted my work … I have a lot of trouble reading the computer screen.* (P2)

#### Early retirement

The deterioration of overall health status was a factor for some in having to cease work resulting in an early retirement due to ill health.

*It’s literally altered every part of my life. My ability to work, my ability to think, my ability to have sex, my ability to be compassionate, everything. There’s not a part of my life that it didn’t massively affect.* (P5)

### Being an employee versus being self-employed

Men who were in paid employment reported that they were entitled to paid sick leave or other leave, which allowed them to focus on their treatment and recovery. They were aware of return-to-work policies at their workplaces and had some autonomy on when and how to return to work. However, not all were confident about how much participation in their usual work would be appropriate. They also felt that the implementation of workplace policies was not always consistent among the management teams of organisations. They reported that they had access to resources such as employee assistance programs at their workplaces, yet they felt that they did not need to access it at the time.

*I had many months of sick leave up my sleeve, so that wasn’t and wouldn’t have been a problem at all. Financially it didn’t affect me at all, so I just took time off …* (P16)

*That was largely they accepted that I could, I would manage my own return to work and my own you know, that I would be the best judge of how much I could do and when I could return to work.* (P3)

*There’s a limit to what you can do from home … working from home was probably ok three quarters of the time, but not all the time … It’s not just those things that department formally provided like the employee assistance program and a return-to-work plan and that sort of thing, that’s great, but as I said you can have a real impediment if your immediate boss isn’t supportive.* (P15)

Being self-employed at the time of diagnosis gave men more control over their work schedule, such as working around treatment sessions, taking time off for tests, treatment, or recovery, and deciding when and how to return to work. Men commented that they had choice of “not travelling anywhere” (P10) or had “flexibility to arrange my work hours to fit in with my treatments” (P13). However, unlike employees, for self-employed men to take time off for treatment or recovery meant loss of income and this was a major concern for them. They remarked that “no work no pay”, “there’s not really any sick leave or annual leave, it comes out of my pocket either way” (P4). The period of not being able to work ranged from weeks to months. Although some of them had personal illness insurance, the coverage only provided temporary and short-term financial relief. Absence from work also meant loss of business opportunities, which resulted in a further loss of income for these men. To minimise the impact of interruption, some men had to give up their plan of easing back into work gradually and returned to work as soon as possible.

*I was offered a job at xxx to do some work … and I had to refuse that ... That would have been twenty thousand dollars’ worth I suppose.* (P10)

*If I stop work that means marketing isn’t done which means work doesn’t come in. It’s not as if I had a choice of diving into work at the beginning (after the surgery), the choice wasn’t there. It was me doing more marketing type stuff, which is still work ….* (P11)

*I had taken out some self-employed sickness insurance many years before … that sort of came extremely handy to give me some sort of minimal income through insurance you know while there was no money coming in … The only burden I had was an influx when the flood gates opened when I returned, which is a little bit tricky in a way because you are still padded up like a cricketer, and you’re not feeling 100% flash, but you just sort of soldier on ….* (P14)

While men who were managing a relatively larger business would have more support from colleagues, it was particularly challenging to maintain business operations for those who were sole or small business owners. In some cases, they were not prepared for a rapid deterioration in their health status and were forced to close the business within a short timeframe and consequently endured financial losses.

*We were able to work around the times when I had my immune system down, I was able to get support from other people within the group to carry out the work that I was going to do.* (P6)

*I guess the nature of my work being a self-employed … you’ve got to be pretty self-motivated and a bit of a self-starter and you’ve kind of got to go out and sort of hustle for work and make things happen. And at various times I’ve just found that really difficult just with the way I’ve been feeling [quite depressed].* (P7)

*[It happened] pretty much within a month … I tried to sell the business; I got someone interested in taking it over but no money. So to keep my customers happy I organised that arrangement [to transfer the business ownership for free].* (P5)

In Australia, people with disabilities are entitled to government social security payments and services. Several self-employed men reported that they found the system was hard to navigate and the application procedure was complicated and stressful. One man found that it “was a big help” when the staff from social services attended the local cancer support service “once a week or once a fortnight” and provided information and assistance on the application procedure. Nonetheless, the financial support they received would not make up for lost income.

*I go to disability job providers and yeah it’s just extremely … no support from them.* (P8)

*They’re not very well set up for people like me who are self-employed, you know you can report income if you’re a wage-earner, but you can’t just sort of report your income fortnightly as a self-employed person … so in trying not to be a burden on the welfare system and trying to be financially independent, I found it’s really difficult.* (P7)

*They gave me the (disability) pension, but they discounted it by how much money she (wife) was earning, so I was getting a hundred dollars a week. And that was very hard to live on.* (P5)

### Personal agency

#### Disclosure of PCa diagnosis

Most men noted they had been open and upfront about their cancer diagnosis at the workplace. They made decisions on whom, when, what and how to talk about the cancer in order to minimise the impact that may be caused by their absence. Some preferred to limit the disclosure to people with whom they had a more established work or business relationship. A few reported that they did hesitate initially and worried that their work would be disadvantaged. Others felt they had little choice but to disclose their condition due to the work settings. Nonetheless, all the men noted that the responses from the workplaces or business partners were positive and “once you let people know, you get a lot more support” (P13).

*I kept them up to date on the whole process … so it was all discussed with my boss, and they scheduled things around me.* (P4)

*It’s sort of a case-by-case basis and it sort of depends on the duration of the work. And I guess the sort of relationship I have with the people involved, whether or not I sort of feel like I need to share that information.* (P7)

*We had a fairly open workplace … people would have noticed if I wasn’t there, and I couldn't say I was sick. I knew I was going to be away for you know a considerable period of time. And I knew that I was going to have to hand my responsibilities to someone.* (P3)

#### Family and wider social support

Men emphasised that families played a key role in their recovery and supporting them to return to work. This included providing emotional support, taking over more home duties and care responsibilities, acquiring information, supporting rehabilitation, and encouraging participants to seek assistance when required. Several men noted that their partners had to take leave or reduce time at work to care for them. By contrast, some partners increased time at work to provide financial support for the family when the man was not able to work or worked less.

Men spoke about how they reached out to the local community or cancer support groups and took part in the group activities, including acquisition of information, sharing information and personal experiences with other men affected by PCa. Some reflected on their experiences and acknowledged the improvement in support services like general PCa information provision, psychological and continence support over the past decade in Australia. Despite the continuous improvement in PCa support, some men felt that some services were less relevant to their individual circumstances. In addition, work related issues or information were rarely discussed in the local support groups and information resources.

*When I was first diagnosed, I started in contact with the Cancer Council of xxx and they provided support for me in terms of sort of discussing with me any problems that I had … talking to people not only in the Cancer Council but also in the prostate community they all were very positive. … So that gave me a lot of confidence in being able to just continue and enjoy the work that I was doing.* (P6)

*Most of the men were a lot older than me and their life circumstances were very different, you know they were retired with grown-up children, and I didn’t really feel like our situations were very comparable and also the advice at the support group I didn’t find particularly useful.* (P7)

*There was a support group in xxx which seem to, if I’d felted the need to, I could have used, but they did seem to be more based on people who had surgery and having those sorts of problems with incontinence.* (P13)

#### Self-care

Most men viewed a positive attitude as a personal strength for managing their health and well-being while continuing work.

*It’s important for me to say I’m a glass half full person, a very optimistic person.* (P9)

Men reported that they made some level of adjustments at work after their PCa diagnosis, such as adopting a gradual return-to-work approach, taking additional breaks and/or temporally reducing working hours, modifying duties, and making adjustments to the workspace. Men exemplified how they managed incontinence at work by limiting certain types of drinks, using pads and/or actively doing pelvic floor exercises. One man explained that he invested in new equipment for his business which enabled him to avoid excessive physical labour and continue working after radical prostatectomy.

Men described how they assessed their own needs or limits and took steps to promote their overall physical and psychosocial functioning, which included active participation in exercises and adoption of a healthy diet, acquisition of information from health professionals or support groups, seeking and using supportive services, such as psychosocial care and continence management. However, some reported that out-of-pocket costs had been a hurdle when accessing services. A few men commented that their peers did not use supportive services because “men do not like talking about problems” (P12). Among those who used these services, not all of them found the services were helpful.

*I put a lot of time and energy into my health, but you know that’s quite expensive. None of those costs are sort of, most of those costs aren’t covered by sort of Medicare or even private health insurance and they’re not tax-deductible.* (P7)

*[The services were] mainly there for social security people … [I] would not be qualified anyway.* (P4)

*My doctor gave me a voucher for half a dozen visits to the psychiatrist because I was emotionally unstable. I wasn’t depressed but I was probably pretty close to it, and I went to see the psychologist half a dozen times and I recall thinking that was a complete waste of time. … All they were interested in finding out was whether I was suicidal or not.* (P5)

*The only thing I did take advantage of for a little while was they had exercise classes, which I attended a few times where I found my own exercise regime was probably just as useful and probably better than the effort of going and using theirs.* (P13)

On the other hand, one man commented that support from health professionals after primary treatment would have helped him build confidence in self-care.

*I would have had one or two consultations with my specialist after the treatment, but you know maybe some more contact with the nurses. Like just to encourage or maybe help me assess my own capabilities. … I was pretty self-motivated, but you know some of that contact might have been you know a bit more …could have added a bit more support.* (P3)

## Discussion

This study explored work-related experiences of men affected by PCa recruited from a community setting. Several important findings emerged from the study. First, although informed decision-making is a core dimension of patient-centred care, it seemed not all of the men in the study felt they were informed appropriately or understood the likely or potential adverse effects of their treatment. As a result, men were less aware of the risks of impaired work ability, and a few found it hard to cope when this occurred. An Australian study [17] with fifty PCa support group leaders reported that men felt they received limited decision support. Improving communication and educating men about their prognosis and treatment options, including possible side-effects, is a priority. Such communication could include a broader focus of men’s quality of life which includes work and employment issues. In the current study, discussion about work-related consequences was found to be largely missing in interactions with healthcare professionals at the treatment decision stage. While men reported that health professionals did not discuss such issues, most men reported survival was prioritised over quality of life, and that they deferred their treatment decisions to health professionals. The communication challenges associated with discussing treatment options and quality of life implications, including impacts on work, are important to recognise. One Dutch study [18] found that 90% of PCa patients did not feel the need to discuss work-related issues and financial consequences of cancer and/or its treatment with a healthcare professional in the hospital. Only 36% of PCa patients reported that healthcare professionals discussed the work-related issues with them and most often during treatment and/or at follow-up. While men prioritise discussions focused on survival, 89% of patients who had conversations about work related issues found it was helpful or somewhat helpful for them. Other related studies in the field [19, 20] have also reported that having such conversations early in the illness trajectory (i.e., before or during treatment) has a positive effect on patients’ work outcomes and mitigates risks of financial difficulties. These findings highlight the importance of two-way communication in which health care professionals provide thorough, effective explanations of general and personalised treatment options and associated side-effects, and initiate discussions with men about the nature of their work, intention to work during and after treatment, and work-related consequences of cancer and its treatment. Men who are encouraged to engage in such conversations and actively seek guidance as early as the treatment decision phase are likely to experience some positive benefits. An integrated health care and work rehabilitation system, such as those implemented in some European countries [21], would facilitate such communication.

For salaried men, sick leave entitlement and being able to negotiate favourable working arrangements allowed them a gradual return to work. Rehabilitation programs were also offered at some workplaces. However, the experiences of self-employed men were notably different. Despite the proportion of self-employed men in the workforce decreasing slightly from 13.2% in 2011 to 10.8% in 2020 in Australia, they have made a substantial contribution to economic growth and society, such as by providing jobs for others [22]. Research on work participation among self-employed cancer survivors has been largely neglected. A limited number of studies using quantitative approaches suggest that self-employed cancer survivors are more likely to continue working during treatment and have less time off work after cancer diagnosis compared to salaried employees [23, 24]. However, they are more likely to experience negative financial consequences and change occupations [24–26]. In addition, they more often than paid employees report poor overall health, lower quality of life and work ability [24, 25]. In accordance with these studies, our findings confirmed that though self-employed men in this study were heterogenous by occupation, size and sector of business, and socio-economic status, most of them faced greater challenges than their salaried counterparts following PCa diagnosis in relation to work participation. Although self-employment offered men freedom and autonomy regarding scheduling and pace of work, self-employed men felt increased pressure to continue to work during treatment or return to work early partially due to financial and business consequences. High financial burden, and difficulties experienced navigating and receiving support from the social welfare system when needed were a concern. The narratives also suggest that reduced work ability and lack of support in the workplace when working alone or in small business increased the risk of business failure. These findings reinforce the need to recognise different aspects of work-related issues, the extent to which the impact of PCa varies in salaried and self-employed men, and the lack of flexibility in the current employment/social support systems. Most interventions to support cancer survivors in returning to work have been developed for salaried employees and would not be appropriate for the self-employed [27]. For example, factors such as income security, social welfare provisions, business insurance, access to financial resources, and government schemes supporting self-employed and small business owners who have serious illnesses may influence the work-related decisions made among self-employed cancer survivors [23]. Further research relating to the policy reforms is needed to support this subgroup to retain work and business.

Men in this study reprioritised their life goals and activities, and most had a strong desire to continue to work or return to work, which was consistent with other studies [6, 7]. However, PCa and its treatment side-effects (e.g., urinary problems, fatigue, depression) had been detrimental to their work capacity and ability and prevented them from working at least for a period of time. Previous research [28, 29] suggests the severity of physical, psychological, and cognitive impairments influences men’s decisions and readiness to work and is associated with work reduction and early retirement. More importantly, such impact could persist over an extended period, as experienced by several men in this study. Longitudinal studies [30–32] show that such health burdens and adjustment could be lengthy and not linear. Indeed, a recent Australian population study [33] estimated that half of all cancer survivors experienced long-term work disability as a result of their cancer or its treatment. Yet research on long-term work-related experiences across different phases of cancer survivorship was scarce. Our findings revealed that it is important to recognise the variability and fluidity across men’s employment arrangements, across the spectrum of survivorship care and offer services to address work disability at each stage. This will require ongoing support to be made available to these men and more flexible adjustment from themselves and their workplaces.

Finally, despite an increase in PCa support initiatives being implemented in Australia over the past two decades, very few men in this study were aware of information and/or resources/services that focus on helping them deal with work-related issues during or after treatments. Growing evidence supports work participation as an essential component of survivorship care [13, 34] and there is an urgent need to develop and promote resources that address this gap. Nonetheless, men in this study have demonstrated varying degrees of personal agency since their diagnosis. Personal agency has been shown to positively enhance self-management behaviours and adjustment of work-related issues in cancer survivors [5, 35]. Men reported positive attitudes towards work and a range of active coping and adjustment strategies to help them deal with general cancer-related and work specific issues. Meanwhile, our finding also revealed that some issues hindered men’s personal agency, such as extra costs for rehabilitation, and lack of self-confidence and individualised support.

Considering all themes, our findings confirm that men’s work-related experiences after PCa diagnosis are multifaceted [7, 12, 13], and factors include the personal (e.g., motivation to work, treatment decision-making, disclosure of cancer, physical and psychological impairments and well-being, process of adjustment, coping mechanism), those relating to employment (e.g., types of employment, workplace policy, income protection), and broader contextual factors (e.g., social policies). Addressing all of these factors requires great cooperation between different stakeholders, including people with cancer and their carers, health and social care professionals, employers, and policy makers [21].

### Strength and limitations

Due to the recruitment setting, our findings reflect only the views of men who attended a PCa support group and were willing to discuss their experiences. These men may be more likely to access information resources and/or peer support, or differ in other ways from those who did not attend these groups. The data may be impacted by recall bias as some participants had been diagnosed for a long time. However, the recruitment strategy enabled us to capture the experiences of a diverse group of men with a broad-spectrum survivorship continuum and demographic features, and in particular included the experiences of self-employed men, and those who live with long-term effects of PCa (more than five years post-diagnosis), and/or experienced cancer progression.

## Conclusions

This study demonstrates that PCa and its treatment have a substantial and persistent impact on men’s working life, and their work participation experience can be diverse and multifaceted. Furthermore, the findings highlighted work-related issues that have not previously been explored among self-employed and long-term PCa survivors. Future studies need to focus on men who face greater challenges and early identification of those that are at high risk of poor work outcomes. While health professionals can play a key role in assisting men to remain at work or to return to work, it is important to recognise and support men’s personal agency in managing their own health and wellbeing, including that of their work life in the survivorship phase. This study also reveals that men’s work experience after PCa diagnosis varies considerably upon contextural factors that go beyond health care. A systematic approach and involvement of stakeholders at all levels are required to support their work participation.

## Data Availability

The datasets generated and/or analysed during the current study are not publicly available to protect the participants’ privacy. De-identified data from this study will be made available on a case-by-case basis from the corresponding author on reasonable request.
